# Big Catch-up vaccination initiative in Central and West Africa: progress, challenges and perspectives- a secondary analysis of aggregated program data

**DOI:** 10.11604/pamj.2026.53.163.49196

**Published:** 2026-04-17

**Authors:** Sylvain Honoré Woromogo, Hadiatou Diallo, Milse William Nzingou Mouhembe, Gilles M'boutiki, Ado Mpia Bwaka, Hilaire Dadjo

**Affiliations:** 1 WHO Intercountry Support Team for West Africa, Ouagadougou, Burkina Faso

**Keywords:** Big Catch-up, zero-dose, under-immunized, immunization

## Abstract

**Introduction:**

WHO and other partners initiated Big Catch-up (BCU) to catch up children aged 12 to 59 months (about 5 years) who have never been vaccinated or are under-vaccinated because of the COVID-19 pandemic. Among the eligible countries are 16 from Central and West Africa. This study aims to analyze challenges and opportunities for this global initiative's success.

**Methods:**

this was a secondary analysis of aggregated program data conducted in 16 countries in Central and West Africa eligible for the BCU. Data were collected through the BCU plans developed by countries, on vaccination coverage for each antigen included, epidemiological surveillance data for vaccine-preventable diseases (VPD) in countries and narrative reports on the implementation of BCU. The proportions of remaining targets to be achieved by country are obtained by dividing the number of children vaccinated in the numerator by the targets for zero doses or under-immunized children in the denominator by antigen. A simple logistic regression was performed to establish relationships between vaccination and the occurrence of vaccine-preventable diseases after COVID-19 and the implementation of BCU.

**Results:**

outbreaks of VPD increase significantly in post-COVID-19 cases and during BCU implementation. All countries except Nigeria still have at least 70% of targets to catch up by December 2025. Two countries are categorized as making good progress, having achieved more than 50% vaccination coverage and DTP and measles: Nigeria and Mali. Cameroon, Côte d’Ivoire and Burkina Faso are making average progress in terms of vaccination coverage of the same antigens between 20-49%.

**Conclusion:**

this initiative helped to curb the occurrence of VPD epidemics. However, the challenges are enormous. Countries must continue to organize further large-scale catch-up rounds and seize every opportunity for vaccination to significantly reduce ZD and Ui and thus VPD epidemics.

## Introduction

The COVID-19 pandemic, with all its corollaries (restrictions on movement, confinement, etc.), has destabilized countries' health systems, leading to a worrying decline in the vaccination coverage of the Expanded Programme on Immunization (EPI) [1-4] and an explosion in the number of unvaccinated and under-vaccinated children worldwide. This decline in coverage has led to an increase of nearly 40% in the number of zero-dose (unvaccinated) children globally, from 13.3 million in 2019 to 18.2 million in 2021 [5]. World Health Organization (WHO) and UNICEF estimates of national immunization coverage (WUENIC) for 2022 found that a total of 25 million children were unvaccinated or under-vaccinated in 2021 (defined as the first and third doses of diphtheria, pertussis and tetanus vaccine). This is 2 million more than in 2020, and 6 million more than in 2019 [5,6]. In order to reduce the number of children who have not received any doses, it is essential to expand immunization services beyond health facilities, in the informal sector, to meet the vaccination needs of children living in underserved places [7,8]. To address the impact of COVID-19 on the performance of the immunization system, WHO, UNICEF, Gavi and IA2030 partners launched a recovery plan called the “Big Catch-up” to address immunization gaps caused by declining immunization coverage during the COVID-19 pandemic [5,9,10]. This initiative has three main objectives: “to reach children who missed vaccination during the period 2019–2022, which was partly due to the pandemic, and provide all missing vaccinations; restore vaccination coverage in 2023 at least to 2019 levels; and strengthening immunization systems as part of primary health care approaches, to improve program resilience and accelerate the achievement of goals and targets of IA2030 and Gavi 5.1” [10]. Most unvaccinated children (or children who have not received any doses) live in hard-to-reach rural areas, urban slums and conflict-affected communities, where health facilities are usually non-existent or difficult to access.

The targets for the big Catch-up are children aged 12 to 59 months who have not received any dose of the vaccine or who are under-vaccinated, to reduce the deficits in immunity in older age groups 13,930,930 missed children (8,102,688 zero-dose and 5,828,242 under-vaccinated) in the Central and West Africa region through 4 major approaches: i) strengthening routine activities in underserved priority areas for catch-up; (ii) periodic intensification of routine immunization (PIRI) such as Child Health Days or Immunization Weeks; (ii) supplementary immunization activities (SIAs); and (iv) outreach and mobile activities in remote communities or a combination of these approaches [10]. The BCU includes a formal monitoring, evaluation and learning framework that focuses on strengthening the monitoring of catch-up immunizations at the level of countries' administrative systems, rapid assessments to correct the course of catch-up activities in real time, and case studies to generate evidence and lessons to guide the long-term implementation of catch-up strategies in the future at country level [10]. His implementation requires the 16 countries to identify challenges, present results, draw lessons learned to participate by the end of 2025 in an evaluation of this initiative. The objective of this study is to show the progress, challenges and prospects of this initiative with the aim of reducing the occurrence of outbreaks of vaccine-preventable diseases.

## Methods

**Type and setting of study:** it was a secondary analysis of aggregated program data that concerned the 16 countries eligible for BCU initiative between 2023 and 2025. Eligible countries called ‘priority countries’ have been endorsed by WHO, UNICEF, the GAVI Alliance, as well as the entire Immunization Agenda 2030 (IA2030) partnership and are distributed as follows: 5 countries in Central Africa (Burundi, Cameroon, Central African Republic, Democratic Republic of the Congo and Chad); 11 countries in West Africa: Benin, Burkina Faso, Côte d’Ivoire, Gambia, Guinea, Guinea-Bissau, Mali, Mauritania, Niger, Nigeria and Togo.

**Description of the data collection procedure:** participants in the study were the WHO focal points for immunization and country-level surveillance to assist with data collection. The study also called on certain partners in immunization at the country level, namely UNICEF, some NGOs, etc.

**Inclusion criteria:** countries that implemented at least one major remedial transition or cycle of activities during the study period were included.

**Exclusion criteria:** countries that have implemented at least one major catch-up or cycle of activities but whose data were not transmitted during the study period.

**General organization of the study:** after the approval of the protocol by the WHO VPD team, the IST/WCA team for BCU provided a briefing to the WHO focal points for vaccination and surveillance as well as the data managers on the modalities for collecting and transmitting data related to the study. WHO consultants “catch up” where they are, collect the data and transmit it to the main investigation team based at IST/WCA in Ouagadougou. IST/WCA data managers helped extract some surveillance and vaccination data and provided their analysis. Data collection and management.

**Data sources:** data were collected through the “Big Catch-up” plans drawn up by the countries; data on vaccination coverage for each antigen included in the “Big Catch-up” through the WUENIC database and country administrative coverage. These data cover the period 2019-2024. Epidemiological surveillance data for countries' vaccine-preventable diseases. These data cover the period 2019-2025. Narrative reports on the implementation of the “Big Catch-up” developed by countries. A template has been developed for this purpose to harmonize data collection.

**Data description:** the “Big Catch-up” plans produce the following data: targets (zero doses and under-vaccinated children); the strategies adopted (routine immunization, PIRI, SIA or a combination of strategies); the objectives of the “Big Catch-up”, the planned demand generation activities, etc. Epidemiological surveillance data produce information on surveillance indicators for each vaccine-preventable disease, case trends and epidemic thresholds, etc. The “Big Catch-up” Implementation Narrative Reports provide evidence on good practices and lessons learned. This data made it possible to: describe the “Big catching-up” profiles of countries; identify good practices that have made it possible to catch up with the targets, as other countries and the next rounds of catch-up. To measure the impact of this strategy on the reduction or not of epidemic episodes.

### Data analysis

The proportions of the remaining targets to be achieved by country are obtained by relating the number of children vaccinated during BCU in the numerator to the zero-dose or under-immunized children targets in the denominator and by antigen. The numerator, the number of children vaccinated is obtained by counting the number of children who have received catch-up vaccinations and recording this information in the BCU tools developed for this purpose. The denominator is derived from a triangular analysis that considers children removed from vaccination registers and a mapping of ZDs carried out by community relays. The denominators for each country have been validated by the members of the Regional Working Group. A comparison of vaccination coverage trends was made according to the objectives set by the countries and the targets achieved to categorize the countries as “good progress”, “medium progress”, “low progress”. We compared trends in vaccine-preventable diseases before and after the “catch-up” using a Chi^2^ test or equivalent. Simple logistic regression at the 0.05 level was applied to determine the relationship between vaccination and the occurrence of vaccine-preventable diseases by specifying Odds Ratios (ORs) and their 95% confidence intervals (CIs).

**Ethical considerations:** the protocol was submitted to the WHO Ethics Committee before the actual start of data collection. We obtained the approval of the countries to use their data. The study is not focused on individuals, so informed consent is not helpful.

## Results

**Trends in vaccine-preventable diseases in the West and Central Africa bloc before and after COVID-19 (for the 16 BCU countries):** in both West and Central Africa, there has been an increase in outbreaks of vaccine-preventable diseases (VPDs) such as measles, yellow fever, diphtheria and neonatal maternal tetanus before and during the implementation of BCU. However, we have observed the beneficial effects of the Big Catch-up for antigens such as measles and tetanus. Table 1 and Table 2 present trends for West and Central Africa. All cases with unknown status are considered unvaccinated (Table 1 and Table 2). We also observed a downward trend in the number of cases of vaccine-preventable diseases after and during the implementation of the catch-up, by age group, except for measles (Table 3).

**Table 1:** trends in vaccine-preventable diseases in the West African bloc before and during the implementation of the Big Catch-up for children zero-doses and under-immunized (for the 11 countries)

MEV	Vaccination status of cases	Number of cases in 2022 - 2023	Number of cases in 2024 - 2025	OR (95 % CI)	P
Yellow fever	Unvaccinated	68	268	2.26 (1.15 - 4.45)	0.0065
	Vaccinated	11	98	-	
Poliomyelitis (cVDVP2)	Unvaccinated	47	13	2.89 (1.91 - 5.55)	< 0.001
	Vaccinated	185	148	-	
Measles	Unvaccinated	18 195	29 288	0.93 (0.89 - 0.97)	< 0.001
	Vaccinated	3 741	5 603	-	
TMN	Unvaccinated	04	47	0.23 (0.06 -0.81)	0.018
	Vaccinated	09	24	-	

**Table 2:** trends in vaccine-preventable diseases in the Central African bloc before and during the implementation of the Big Catch-up of children zero-doses and under-immunized (for the 05 countries)

MEV	Vaccination status of cases	Number of cases in 2022 - 2023	Number of cases in 2024 - 2025	OR (95 % CI)	P
Yellow fever	Unvaccinated	31	101	2.63 (1.09 - 6.34)	0.019
	Vaccinated	07	60	-	
Poliomyelitis (cVDVP2)	Unvaccinated	42	18	2.26 (1.23 - 4.14)	0.004
	Vaccinated	133	129	-	
Measles	Unvaccinated	19 165	31 288	0.88 (0.85 - 0.93)	< 0.001
	Vaccinated	3 941	5 693	-	
TMN	Unvaccinated	-	-	-	-
	Vaccinated	-	-	-	

**Table 3:** distribution of cases of vaccine-preventable diseases by age group before and during the catch-up implementation period in West Africa (11 countries)

MEV	Age range		Year 22-23	Year 24 - 25
Yellow fever	0-4 years	Vaccinated	04	52
		Unvaccinated	03	128
	5 years +	Vaccinated	07	46
		Unvaccinated	65	139
Poliomyelitis (cVDVP2)	0-4 years	Vaccinated	174	142
		Unvaccinated	42	13
	5 years +	Vaccinated	11	06
		Unvaccinated	05	00
Measles	0-4 years	Vaccinated	2481	3957
		Unvaccinated	12448	19287
	5 years +	Vaccinated	1260	1635
		Unvaccinated	5787	9962

**Strategies for implementing catch-up activities:** the analysis of the implementation plan for the activities of BCU made it possible to identify several strategies for achieving the targets to be caught up before the end of December 2025. In the second half of 2025, each of the 16 countries has implemented at least one passage of BCU. Table 4 gives an overview of these strategies for the 16 countries of the Central African and West African bloc selected for BCU.

**Table 4:** strategies adopted by the 16 countries of the Central Africa West Africa bloc selected for Big catch-up by December 2025

Country	Type of activity	Scope
Benin	Integration with the measles and other SIA vaccination campaign	The whole country
Burkina Faso	PIRI	The whole country
Burundi	PIRI	The whole country
Cameroon	PIRI	80 priority districts
Central African Republic	PIRI	The whole country
Ivory Coast	PIRI	113 priority districts
Democratic Congo	PIRI	7 priority provinces
Gambia	PIRI	The whole country
Guinea	PIRI	The whole country
Guinea Bissau	PIRI	The whole country
Mali	PIRI	44 priority districts
Mauritania	Routine immunization	The whole country
Niger	PIRI	33 districts identified
Nigeria	PIRI	30 targeted states
Chad	PIRI - Integration with additional PCV and yellow fever vaccination activities	The whole country
Togo	PIRI	The whole country

**Proportion of zero-dose and under-vaccinated children caught up in the bloc (16 BCU countries):** the situation of ZD and Under-immunized children is presented in Table 5. In the second half of 2025, all the countries of the bloc have achieved at least one passage of BCU. Nigeria has a low proportion of ZD and Ui of less than 30% to catch up by December 2025. Mali has a proportion of ZDs at 63% and 45% for Ui. The remaining countries have more than 70% of their targets to catch up with by the end of December 2025.

**Table 5:** targets to be vaccinated and remaining proportion to be vaccinated by December 2025 in Central and West Africa as part of the Big Catch-up in the second half of 2025

Country	ZD Targets	Under-vaccinated targets	Nb ZD vaccinated	Nb under-vaccinated vaccinated	Proportion of ZD remaining at the end of December 2025 (%)	Proportion of under-vaccinated people remaining by the end of 2025 (%)
Benin	247 742	380 662	28 124	28 482	89	93
Burkina Faso	223 260	223 260	31 833	51 604	86	77
Burundi	155 290	160 051	2 778	3 172	98	98
Cameroon	299 647	299647	107 683	110 595	64	63
Central African Republic	308 964	438729	2 422	3 320	99	99
Ivory Coast	436 897	733 147	65 598	104 390	85	86
Democratic Congo	1 795 598	3 487 327	306 581	352 668	83	90
Gambia	33 199	46 256	2 317	2 095	93	95
Guinea	492 136	686 400	57 595	74 942	88%	89
Guinea Bissau	24 844	37 708	1 408	1 267	94%	97
Mali	74 642	227 007	121 771	125 525	-63	45
Mauritania	77 270	105 160	3 077	2 756	96	97
Niger	279 186	723 994	54 398	58 247	81	92
Nigeria	2 437 427	2 902 700	1 920 043	2 422 091	21	17
Chad	325 937	823 157	53 215	54 162	84	93
Togo	69 672	94 518	2 988	4 599	96	95

### Dynamics of antigen vaccination coverage for the Big Catch-up

**Figure 1:**
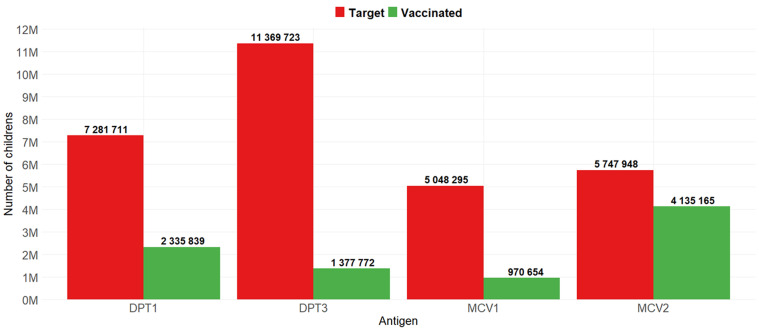
evolution of vaccination targets achieved and number of children remaining to be vaccinated by December 2025 by antigen in the second quarter of 2025 for the 16 Big Catch-up countries in Central and West Africa

**Figure 2:**
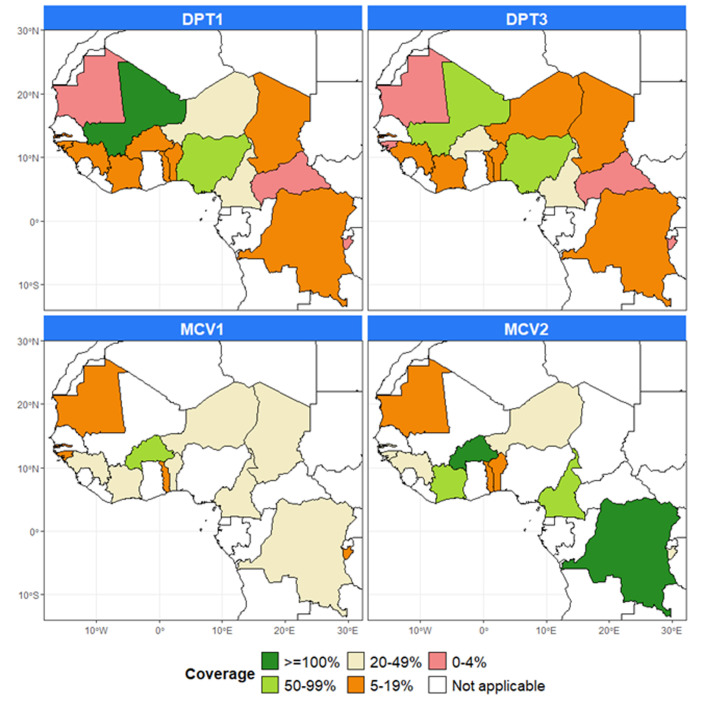
analysis of progress in achieving vaccination coverage for DTP, measles antigens for the 16 Big Catch-up countries in the second half of 2025

Countries have administered all the antigens selected for BCU. Since the beginning of the catch-up, just over a third (DTP1: 32.9%) of children who have not received any dose and less than a quarter (DTP2: 12.6%) of under-immunized children have been affected. Nearly three-quarters of children have received the second dose of measles vaccine (children over 2 years of age). Twenty-eight percent (28%) of IPV doses and 13% of bOPV doses were administered to children, respectively (Figure 1). In the second year of 2025 alone, 424,339 children who had not received any dose and 2,020,752 children who had received a single dose were caught up. Nigeria, DRC and Niger have made remarkable efforts in vaccinating more than 100,000 children. In the second half of 2025, two countries are categorized as making good progress, having achieved more than 50% vaccination coverage and DTP and measles: Nigeria and Mali. Cameroon, Côte d'Ivoire and Burkina Faso are making average progress in terms of vaccination coverage of the same antigens between 20-49% (Figure 2).

## Discussion

### Trends in vaccine-preventable diseases

The COVID-19 pandemic has led to outbreaks of vaccine-preventable diseases such as measles, yellow fever, polio and others in the 16 countries in West and Central Africa targeted by the Big Catch-up Initiative. These outbreaks have continued to rage even after the start of the Big Catch-up in the 16 countries of the Central and West African bloc targeted by this strategy. Aside from the COVID-19 pandemic, vaccine-preventable diseases are both opportunities and challenges in most countries around the world [11,12]. Measles outbreaks remain a major public health problem even during the implementation of the initiative. They are the prerogative of many countries outside the West and Central African bloc [13-16]. The introduction of the second dose of the measles vaccine by most of these countries will help boost children's immunity. Unvaccinated children are more likely to catch VPDs than vaccinated children, whether in the post-COVID-19 period or during the implementation of the Big Catch-up. We have observed several cases of VPD during the implementation period of BCU compared to the post-COVID-19 period. This is due not only to the accumulation of the number of zero-dose and one-time vaccinated children and to the strengthening of disease surveillance at the country level. However, it would be advisable that the calculation of ORs is based on aggregated national or bloc-level data for vaccinated vs. unvaccinated groups. This makes the OR highly susceptible to the Ecological Fallacy.

### Importance of Big Catch-up

Countries have developed plans to implement BCU with targets ranging from 20 to 100 percent. Overall, in the second half of 2025, countries caught up with many zero-dose and under-vaccinated children. But many more remain to be caught up and the remaining proportions to be caught vary from 21 to 99% and 17 to 97% for ZD and Ui respectively. Nigeria and Mali have the lowest proportions to catch up; Mali set a target of catching up to 20% of ZD and Ui initially because of security challenges but vaccinated more children than expected. The use of strategies has made it possible to obtain these results in the example of several countries [17-19]. But the observation is that much remains to be done for the deadline for the end of BCU scheduled for December 2025. Countries are encouraged to develop and implement a plan to accelerate catch-up and seize every opportunity to vaccinate targets. The opportunities identified underway are scheduled vaccination campaigns, pertussis and diphtheria epidemics for which some countries are preparing to respond. Specifically, all countries still have a high proportion of children to catch up with per antigen. The high proportion of children caught up with the second dose of the measles vaccine is explained by the multiple response campaigns against measles epidemics and by the second year of life when this dose is administered.

### Challenges and opportunities

The challenges of reducing children’s ZD and Ui remain permanent for the 16 countries [5]. The analysis of countries' narrative activity reports shows that they are facing not only the challenges of implementing BCU rounds but also routine immunization activities, not to mention reactive vaccination campaigns or supplementary immunization activities. Another challenge related to precedents is that countries tend to forget that these challenges can turn into opportunities to catch up with targeted children. Another final challenge for countries is the target of BCU that must be achieved absolutely (the children of 12-59 months) [9] so that they do not leave the calendar of BCU and maintain the outbreaks of VPD. Countries such as the DRC, Niger, Burkina Faso, Mali, the Central African Republic and Chad are facing security challenges and are developing significant strategies to catch up with children. Conflicts and other disasters have a negative impact on vaccination coverage and infectious diseases [20-22].

## Conclusion

Countries in the West and Central African bloc targeted by the BCU initiative experienced outbreaks of vaccine-preventable diseases before and during the initiative, far more among unvaccinated children than among vaccinated children. This initiative has helped to slow down the occurrence of measles epidemics and neonatal maternal tetanus cases. Several children who have never been vaccinated or are under-vaccinated have been caught during this initiative. Countries should continue to hold more catch-up rounds and seize every opportunity for immunization to significantly reduce the number of unvaccinated children and thus VPD outbreaks.

### What is known about this topic

- Thousands of children were taken out of the vaccination schedule and escaped vaccination when the COVID-19 pandemic broke out;
- Countries in Central and West Africa have experienced outbreaks of vaccine-preventable diseases because of the increase in unvaccinated and under-vaccinated children;
- The catch-up initiative is complementary to routine immunization at the country level.

### What this study adds

- The BCU has made it possible to vaccinate many unvaccinated and under-vaccinated children registered after the outbreak of the COVID-19 pandemic, mainly with the help of strategies such as the periodic intensification of routine immunization, this initiative enabled children who were no longer covered by their countries' vaccination schedules to be vaccinated;
- The Big Catch-up should continue by incorporating other routine immunization opportunities to significantly reduce the number of zero-dose children and thus reduce outbreaks of vaccine-preventable diseases;
- The persistence of outbreaks of vaccine-preventable diseases is due to the accumulation of unvaccinated and under-vaccinated children.
